# Mobile Phone Text Message Reminders to Improve Vaccination Uptake: A Systematic Review and Meta-Analysis

**DOI:** 10.3390/vaccines12101151

**Published:** 2024-10-08

**Authors:** Gail Erika Louw, Ameer Steven-Jorg Hohlfeld, Robyn Kalan, Mark Emmanuel Engel

**Affiliations:** 1 Cape Heart Institute, Department of Medicine, Faculty of Health Sciences, University of Cape Town, Cape Town 7925, South Africa; louwgail@gmail.com (G.E.L.); robyn.kalan@gmail.com (R.K.); 2 Health Systems Research Unit, South African Medical Research Council, Tygerberg 7501, South Africa; ameer.hohlfeld@mrc.ac.za; 3 South African Cochrane Centre, South African Medical Research Council, Tygerberg 7501, South Africa

**Keywords:** vaccination, text message reminders, vaccination uptake, vaccination coverage

## Abstract

Introduction: Mobile phone text message reminders (MPTMRs) have been implemented globally to promote vaccination uptake and recall rates. This systematic review evaluated the effectiveness of MPTMRs on vaccination recall rates. Methods: We included randomized controlled trials of caregivers of children, adolescents, or adults who received MPTMRs for improving vaccine uptake and recall visits. We searched the Cochrane Central Register of Controlled Trials (CENTRAL), PubMed, and Scopus to identify relevant studies published up to 24 January 2024. We used Cochrane’s Risk of Bias tool to assess the included studies and reported the results as risk ratios with 95% confidence intervals, using a random effects model. Results: We identified 25 studies for inclusion. All studies were assessed as having a low risk of bias. The evidence supports MPTMRs for improving vaccination uptake compared to usual care (RR = 1.09 [95%CI: 1.06, 1.13], I^2^ = 76%). Intervention characteristics, country setting, country economic status, and vaccination type had no bearing on the effectiveness of the intervention. Conclusions: MPTMRs have a positive effect, albeit relatively small, on vaccination uptake. These findings may assist public health practitioners, policymakers, and vaccine researchers in evidence-based decision making that focuses on MPTMRs and their impact on vaccination coverage.

## 1. Introduction

Vaccine-preventable diseases remain a global public health concern despite vaccine availability and vaccine access. Increasing vaccination coverage has been prioritized by the World Health Organization (WHO) to decrease the occurrence of morbidity and mortality from vaccine-preventable diseases. Researchers define vaccination coverage generally as the proportion of individuals who have received a specific vaccine within a defined population [1]. Recent reports demonstrated a significant decrease in global vaccination coverage due to the negative impact of the coronavirus disease 2019 (COVID-19) pandemic on critical health services [2,3]. The WHO/UNICEF estimates of national immunization coverage reported that 25 million children were unvaccinated or incompletely vaccinated, with a reduction in vaccination coverage from 86% in 2019 to 81% in 2021 [2]. These findings have a significant impact on achieving sustainable development goals that focus on promoting and ensuring health and well-being at all ages through vaccination [4].

**Figure 1:**
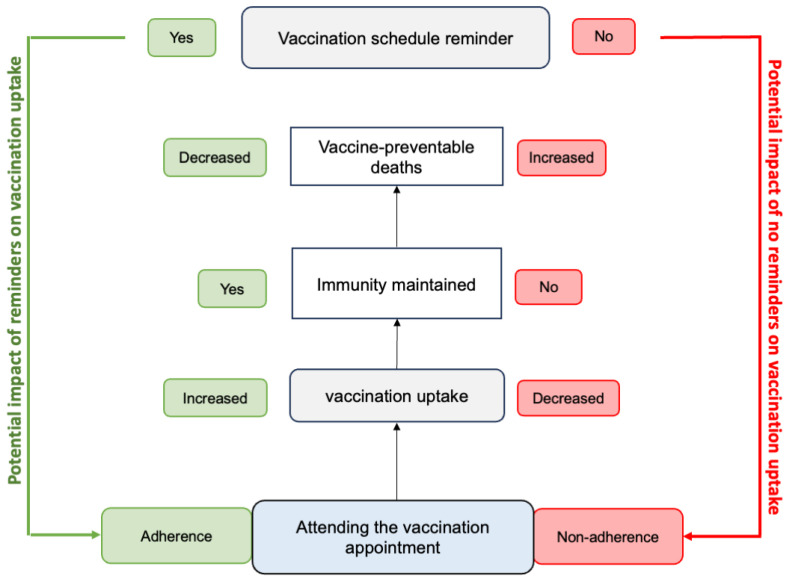
Interplay among factors that influence vaccination uptake.

Timely vaccination and up-to-date vaccination schedules are essential factors that promote maintaining immunity and limiting vaccine-preventable deaths [1,5]. To facilitate the development of country-specific vaccination schedules and promote the on-time administration of vaccines, the WHO has provided guidance on defined vaccination schedules and intervals [6,7]. Figure 1 represents the interplay among factors that can influence vaccination uptake that would facilitate a decrease in vaccine-preventable deaths. It presents the implications of adherence/non-adherence on vaccine-preventable deaths. Maintaining the decline in vaccine-preventable deaths is dependent on vaccination uptake, defined as the “number of individuals that have received a specified vaccine dose(s)” [1]. This, in turn, depends on the scheduling and on-time attendance of the vaccination appointment [8,9,10]. These appointments are often missed and lead to either missed or late vaccinations, resulting in lower vaccination uptake, which could lead to an increase in vaccine-preventable deaths (Figure 1). Various studies have reported that socio-demographic factors such as age and educational level affect adherence to vaccination appointments [11,12].

Similarly, reports have shown that socio-economic status affects vaccination uptake, with low-income households demonstrating lower adherence to vaccination appointments, resulting in lower vaccination coverage [13,14]. Some studies also reported that lack of basic knowledge of the caregiver or adult on the importance of vaccinations, poor service delivery, religious beliefs, and access to health facilities are factors that negatively affect adherence to vaccination schedules and subsequently vaccination coverage [15,16,17,18]. Given these factors, exploring accessible, innovative technologies that can be implemented on a global scale is essential to improve vaccination coverage both regionally and globally.

In 2012, the WHO launched the Global Vaccine Action Plan (GVAP) 2011–2020, with the vision to facilitate an increase in global vaccination coverage [19]. This plan was guided by six principles, including (1) country ownership, (2) shared responsibility and partnership, (3) equity, (4) integration, (5) financial sustainability, and (6) innovative research and development [19]. The goal of GVAP was to achieve 90% national and 80% district level vaccination coverage of DTP3 by 2020 [19]. National vaccination coverage goal of 90% was achieved in 125 countries, while only 57 countries reported a 80% coverage in the three doses of the combined diphtheria, tetanus toxoid, and pertussis (DPT3) vaccine on a district level [20]. Although GVAP did not meet all its goals, it laid the foundation for a solid framework for the Immunization Agenda 2030, which aims to (1) reduce the number of unvaccinated children by 50%, (2) achieve 90% coverage for childhood vaccinations, and (3) achieve 500 introductions of new or under-utilized vaccines in low- and middle-income countries [21]. This framework includes the following seven strategic priorities (1) immunization programs for primary health care and universal health coverage, (2) commitment and demand, (3) coverage and equity, (4) life-course and integration, (5) outbreaks and emergencies, (6) supply and sustainability, and (7) research and innovation [21].

It is evident that improving vaccination coverage would require a substantial sustained global, national, and regional effort and innovative strategies to implement and optimize health systems in target populations. A recent study demonstrated that modifiable factors, such as a lack of maternal knowledge on childhood vaccinations, schedules, and side effects as well as maternal attitude toward vaccination, negatively impacted vaccination uptake in Africa [22]. For this reason, innovative strategies and interventions, such as mobile health and digital health, in addition to mobile phone technology and social media platforms, are being developed to decrease vaccine misinformation and hesitancy and increase vaccination coverage [23,24,25]. Various studies have shown the efficacy of text message reminders either alone or in combination with other interventions, such as postcards, auto dialer calls, and letters, in improving vaccination coverage in defined target populations for various diseases and in different clinical and country settings [26,27,28]. This indicates that text message reminders and recall have the potential to facilitate behavior changes, leading to adherence to vaccination schedules by reminding caregivers of infants, adolescents, and adults of scheduled vaccination appointments and providing the required encouragement to ensure timely attendance to improve vaccination uptake.

A recent systematic review demonstrated that personalized text message reminders for COVID-19 vaccination appointments increased vaccination uptake [29]. However, limited published studies exist that synthesize all available scientific evidence assessing the effectiveness of mobile phone text message reminders (MPTMRs) on vaccination coverage, irrespective of geographic location, population, or disease. This systematic review sought to assess the most recent and best scientific evidence evaluating the efficacy of MPTMRs as an intervention to improve vaccination uptake.

## 2. Materials and Methods

The findings in this systematic review and meta-analysis were reported according to the Preferred Reporting Items for Systematic Review and Meta-Analysis (PRISMA) guidelines [30] and guidelines outlined in the Cochrane Handbook of Systematic Reviews for Interventions [31]. No formal institutional review board approval was required for this study since it does not involve human participants.

### 2.1. Review Question

What is the effectiveness of mobile phone text message reminders (MPTMRs) in improving vaccination recall in children, adolescents, and adults?

### 2.2. Study Outcome

This study assessed vaccination recall as the outcome including subgroup analysis based on the nature of the intervention, country’s economic status, study setting, and vaccination type.

### 2.3. Eligibility Criteria and Search Strategy

The study eligibility criteria for inclusion were pre-defined (Table S1). Briefly, studies were eligible if they were randomized controlled trials (RCTs) of caregivers of children, adolescents, or adults who received MPTMRs as an intervention. The intervention needed to include information provided by short messaging service (SMS), Telegram, WhatsApp, Facebook Messenger, or any multimedia application that uses an instantaneous alert delivered to caregivers of infants, children, adolescents, or adults (including women that were pregnant or breastfeeding) that required a follow-up dose(s) in a routine vaccination schedule or booster vaccinations for any vaccine-preventable disease. Our comparator interventions included usual care, which encompassed, but was not limited to, written appointment reminders on appointment cards or immunization cards, verbal reminders of the next appointment, autodial telephone reminders, text messages with health education content, and no appointment reminders at the health care facilities. Studies were excluded if it did not include a comparator group or if the comparator group was not usual care.

A comprehensive strategy was developed and applied to search and subsequently identify relevant studies (Table S2). This search strategy included key words such as “SMS” and “text reminder” in addition to medical subject heading (MeSH) terms in various combinations such as “immunization”. The search strategy was independently adapted and modified accordingly for searches in PubMed, Cochrane Central Register of Controlled Trials (CENTRAL), and Scopus performed by two investigators (GL and AH). The reference lists of relevant publications identified through the database search were assessed to identify additional studies for full-text eligibility assessment. We screened all studies published until 24 January 2024.

### 2.4. Study Identification and Selection

The database search was independently conducted by two authors (GL and AH), and publications retrieved from the search were uploaded into Rayyan, a semi-automated review screening tool to facilitate the screening process [32]. Duplicate publications were excluded. Both reviewers independently applied the inclusion criteria. Titles and abstracts were screened for relevance. The results between the two independent reviewers were compared; discrepancies were resolved by discussion. Subsequently, GL obtained the full-text publications of potentially eligible studies, and GL and AH independently evaluated each publication for full-text eligibility using the pre-defined inclusion and exclusion criteria (Table S1). The results between the two authors were compared, and discrepancies were resolved by discussion. The reason for exclusion is tabulated in the table of excluded studies (Table S3).

### 2.5. Data Extraction and Management

From studies deemed to be eligible, GL and AH extracted, using a standardized data extraction form, detailed information, including intervention and control characteristics, age category of participants, country and settings where the studies were conducted, and details on the type of vaccines including vaccination schedules. Information on visit/dose-specific vaccination coverage in the intervention and control groups in addition to details on incentives provided to the participants were extracted.

Disagreements regarding data extracted were resolved by discussion until 100% agreement was achieved. All relevant data were entered into The Cochrane Collaboration Review Manager version 5.4.1 [33] software, and a second author conducted a quality control check to ensure no data entry errors occurred.

### 2.6. Assessment of Risk of Bias of Included Studies

GL and AH assessed the risk of bias of each RCT as well as the respective protocols and trial registry records by independently applying the Cochrane Collaboration’s tool for assessing risk of bias in randomized trial [34]. The risk of bias criteria assess bias arising due to (1) random sequence generation (selection bias), (2) allocation of concealment (selection bias), (3) blinding of participants and personnel (performance bias), (4) blinding of outcome assessment (detection bias), (5) incomplete outcome date (attrition bias), and (6) selective reporting (reporting bias) [34]. Following this independent assessment, any discrepant judgements for risk of bias between the two authors were resolved by discussion between the two authors or by an impartial third author where the disagreements persist. We contacted authors of the included studies when missing data or incomplete information was identified.

### 2.7. Measures of Effect

Risk ratio (RR) and mean differences were calculated for dichotomous outcomes and continuous outcomes, respectively.

### 2.8. Data Synthesis

Data analysis was conducted as outlined in the Cochrane Handbook for Systematic Review of Interventions [31]. Meta-analysis was done on the data considered homogenous using The Cochrane Collaboration Review Manager version 5.4.1 [33] software to produce synthesis of the treatment effect together with the respective 95% confidence intervals (95%CIs). Summary effect sizes were estimated using the random-effects model. We conducted analyses on an intention-to-treat basis for all outcomes.

Clinical heterogeneity of the included studies was assessed by evaluating variability in participants, interventions subtypes, and study outcomes. Heterogeneity in methods of the included studies was assessed by evaluating variability in risk of bias. Descriptive statistics were used to characterize both clinical and methodological heterogeneity using the I^2^ statistic.

#### 2.8.1. Subgroup Analysis

Subgroup analysis was done to assess the effect of intervention type (text messaging only vs. text in addition to another component, e.g., appointment card reminders, standard verbal counselling, educational videos, and routine health education), country’s economic status (low- and middle-income countries (LMICs) vs. high-income countries (HICs)), study setting (urban vs. other), and vaccination types (early childhood vaccination vs. HPV vs. other) on the results of the meta-analysis.

#### 2.8.2. Sensitivity Analysis

The effect of excluding studies with a high risk of attrition bias on the intervention effect was determined.

### 2.9. Risk of Publication Bias Assessment

We assessed publication bias by creating a funnel plot using The Cochrane Collaboration Review Manager version 5.4.1 software and plotting the standard errors of log RRs against RRs [33,35].

## 3. Results

### 3.1. Study Identification and Selection

**Figure 2:**
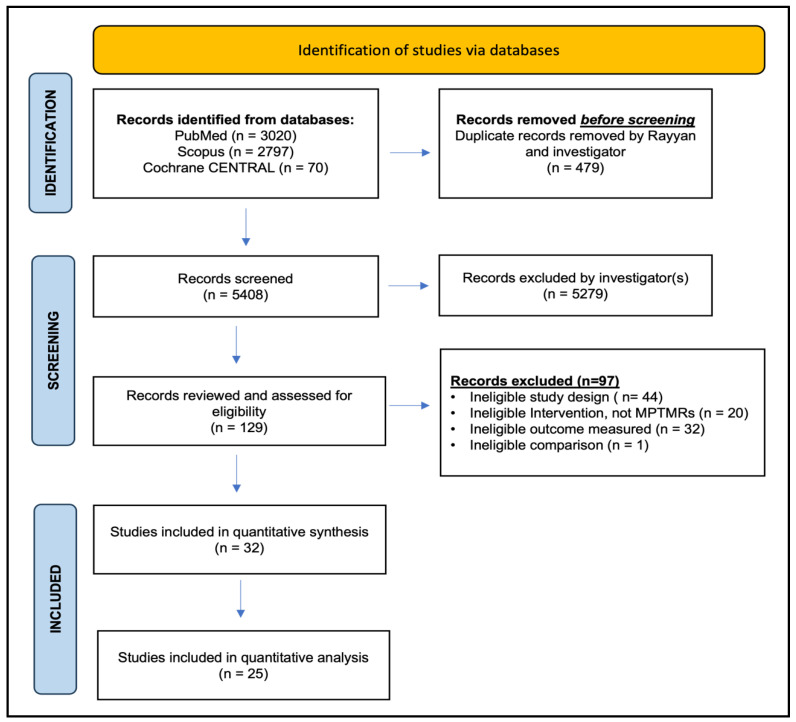
PRISMA diagram of the studies identified in the systematic literature search.

The search identified 5887 publications from the databases search, comprising studies from PubMed (n = 3020), Scopus (n = 2797), and Cochrane CENTRAL (n = 70) (Figure 2). Articles were managed using the semi-automation tool Rayyan.ai [32]. In total, 479 duplicates were removed and the titles, and abstracts of the remaining 5408 publications were screened by GL and AH based on the exclusion criteria outlined in Table S1. Most studies (n = 5279) were excluded. Following this screening process, we selected 129 publications eligible for full-text screening and subsequently identified 32 studies that met the inclusion criteria (Table S1) for data extraction and quantitative synthesis.

The included studies were characterized, with six studies conducted in a rural setting [36,37,38,39,40,41], 22 in an urban setting [42,43,44,45,46,47,48,49,50,51,52,53,54,55,56,57,58,59,60,61,62], and four in a combination of semi-urban and rural or urban and suburban settings [63,64,65,66] (Table S4). The majority of the studies were conducted in the USA (n = 17) [36,40,41,42,43,44,46,47,48,49,52,55,58,60,62,65,67], followed by Africa (n = 6) [37,38,51,56,61,63], Australia (n = 4) [39,42,45,50], and the countries of Pakistan (n = 2) [54,59], Guatemala (n = 2) [57,64] and India (n = 1) [66] (Table S4). Of the 32 studies, seven [37,40,41,48,52,53,63] were excluded from further quantitative analysis since the outcome data were not stratified by the intervention type; thus, 25 studies were included for risk of bias assessment and meta-analysis (Table S4).

Studies were excluded from quantitative synthesis for the following reasons: ineligible study design (n = 44) [68,69,70,71,72,73,74,75,76,77,78,79,80,81,82,83,84,85,86,87,88,89,90,91,92,93,94,95,96,97,98,99,100,101,102,103,104,105,106,107,108,109,110,111], ineligible intervention, not MPTMRs (n = 20) [67,112,113,114,115,116,117,118,119,120,121,122,123,124,125,126,127,128,129,130], ineligible outcome measured (n = 32) [128,131,132,133,134,135,136,137,138,139,140,141,142,143,144,145,146,147,148,149,150,151,152,153,154,155,156,157,158,159,160], and ineligible comparison (n = 1) [161] (Table S4).

### 3.2. Risk of Bias Assessment

**Figure 3:**
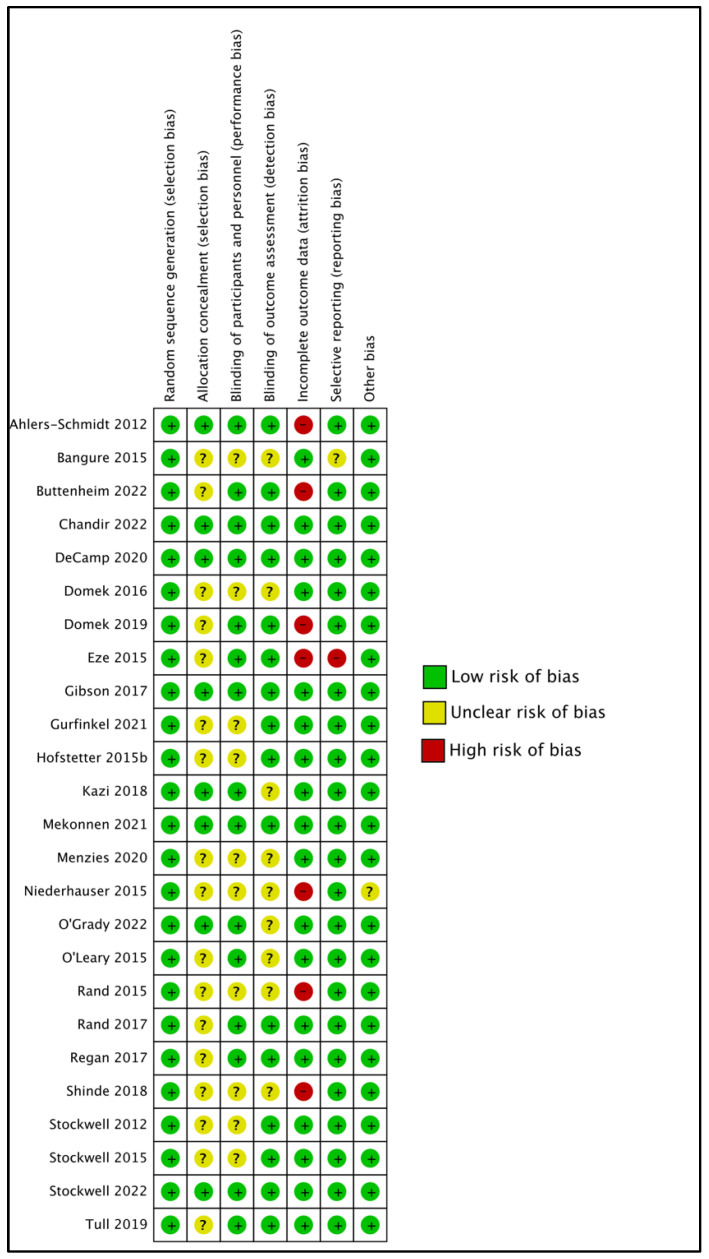
Risk of bias summary of included studies [36,38,39,42,43,44,45,46,47,49,50,51,54,55,56,57,58,59,60,61,62,64,65,66,152].

Randomization sequence generation: All included studies were judged as having a low risk of selection bias with regards to randomization sequence generation (Figure 3 and Figure S1). 

Allocation concealment: An unclear risk of bias for allocation concealment was concluded for most studies (n = 17; 68%).

Blinding: Blinding was assessed as a low risk of bias for most studies (n = 17; 68%). 

Allocation concealment: The majority of studies (n = 17; 68%) were deemed as having an unclear risk of selection bias, with the remaining studies (n = 8; 32%) showing a low risk of selection bias [36,38,39,51,54,58,59] (Figure 3 and Figure S1).

Detection bias: The majority of studies (n = 16; 64%) showed low risks of detection bias where the outcome was assessed by data retrieval and the study analysts were blinded to the group assignments [43]. The remaining studies (n = 9; 36%) showed unclear risks of detection bias because these studies did not provide information on blinding of the assessment outcome (Figure 3 and Figure S1).

Incomplete outcomes: The majority of RCTs had a low risk of bias for incomplete outcome data reporting (n = 18; 72%) and selective reporting (n = 23; 92%) (Figure 3 and Figure S1).

Attrition bias: High attrition bias was observed in seven studies (28%) [36,47,49,56,64,66] (Figure 3 and Figure S1).

All but one study [56] reported a well-defined statistical analysis plan, thereby indicating a high reporting bias (Figure 3 and Figure S1). Additionally, all studies provided reasons for LTFU, which included, e.g., participants moved (out of state) or switched to a different clinic, study staff were unable to contact participants, telephone number disconnected, wrong telephone number, or participants died. Lastly, all but one study [49] showed an unclear risk of other biases (Figure 3 and Figure S1), and this was challenging to assess due to the small sample size.

### 3.3. Quantitative Data Synthesis

**Figure 4:**
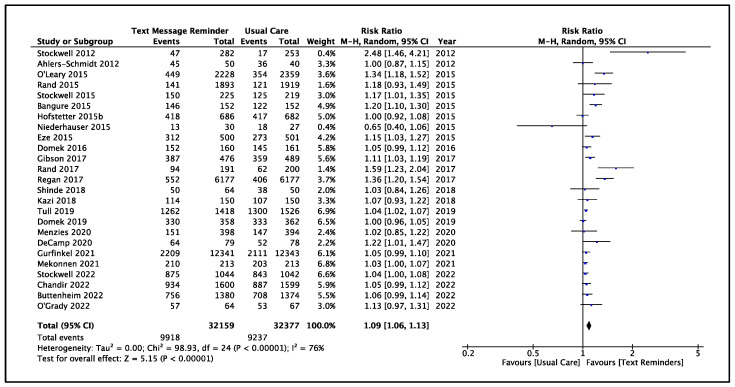
Meta-analysis of data from included studies for vaccination recall after the enrolment visit [36,38,39,42,43,44,45,46,47,49,50,51,54,55,56,57,58,59,60,61,62,64,65,66,152].

Twenty-five studies (n = 64,536 participants) were considered for quantitative synthesis of evidence regarding vaccination recall. Pooled data favored MPTMRs for vaccination recall compared to usual care (RR = 1.09 [95%CI: 1.06, 1.13]; I^2^ = 76%) (Figure 4, Table 1). Given the substantial heterogeneity among the study results, we conducted a sensitivity analysis, omitting studies of poor quality (n = 6) [44,45,46,49,58,65] that maintained the effect (pooled RR = 1.05 [95%CI: 1.03, 1.07]; I^2^ = 33%) (Figure S2). A meta-analysis conducted by excluding the results of studies with a high risk of attrition bias (n = 7) [36,47,49,56,60,64,66] produced similar results (pooled RR = 1.11 [95%CI: 1.07, 1.15]; I^2^ = 79%) (Table 1, Figure S3). A table summarizing the findings is provided in the Supplementary Material (Table S5).

### 3.4. Subgroup Analysis

#### 3.4.1. Intervention Characteristics

The subgroup of MPTMRs with additional components had a similar effect on vaccination recall (RR = 1.10 [95%CI: 1.04, 1.16]; I^2^ = 83%) compared to the subgroup assessing the effect of MPTMRs alone (RR = 1.09 [95%CI: 1.04, 1.15]; I^2^ = 71%). The test for subgroup effect based on intervention characteristics demonstrated no statistically significant effect and no heterogeneity between the results of the two subgroups (I^2^ = 0%) (Table 1, Figure S4). This suggests that the intervention characteristics do not modify the effect of MPTMRs compared to usual care.

#### 3.4.2. Country Setting

Subgroup analysis based on country setting showed no effect with no heterogeneity between results in the subgroups (I^2^ = 0%) (Table 1, Figure S5). Within the urban subgroup, the results show an effect in favor of MPTMRs (RR = 1.10 [95%CI: 1.06, 1.14]; I^2^ = 73%). Rural and/or suburban and/or semi-urban also shows an effect in favor of MPTMRs; however, there was no significant difference (RR = 1.10 [95%CI: 0.97, 1.24]; I^2^ = 89%) (Table 1, Figure S5).

#### 3.4.3. Country Economic Status

The results show an effect in favor of MPTMRs regardless of a country’s income status. MPTMRs, when analyzed in the LMIC subgroup (RR = 1.07 [95%CI: 1.03, 1.11]; I^2^ = 65%), were slightly less effective than in the HIC subgroup (RR = 1.12 [95%CI: 1.06, 1.18]; I^2^ = 79%), with substantial heterogeneity observed in both subgroups (Table 1, Figure S6). The test for subgroup differences in country economic status shows that there is no statistically significant effect between the LMIC and HIC subgroups; moderate heterogeneity between results in the subgroups (I^2^ = 42.3%) was observed (Table 1, Figure S6). The HIC subgroup had more participants (57,186 participants) who contributed to the subgroup analysis compared to the LMIC subgroup (7350 participants), which may indicate that the analysis may not be able to detect subgroup differences.

#### 3.4.4. Vaccination Type

The test for subgroup differences in vaccination type shows that there is no statistically significant effect among the vaccination types; however, moderate heterogeneity between results in the subgroups (I^2^ = 38.6%) was observed (Table 1, Figure S7). In all three subgroups, the results show an effect in favor of MPTMRs with RR = 1.07 [95%CI: 1.03, 1.11] observed in the early childhood vaccination subgroup, RR = 1.17 (95%CI: 1.05, 1.30) observed in the HPV subgroup, and RR = 1.14 [95%CI: 1.01, 1.28) observed in the other vaccination subgroup that comprised studies that investigated seasonal influenza vaccination recall. In addition, substantial heterogeneity was observed for the results in the early childhood vaccination subgroup (I^2^ = 63%), HPV subgroup (I^2^ = 86%) and the other vaccination type subgroup (I^2^ = 88%) (Table 1, Figure S7).

### 3.5. Risk of Publication Bias Assessment

Assessment of the funnel plot indicates a symmetrical plot that suggests the absence of publication bias (Figure S8).

## 4. Discussion

### 4.1. Summary of Main Findings

This meta-analysis of 25 studies presents the most recent available evidence on the effectiveness of MPTMRs on vaccination uptake in adolescents, children, and adults in all clinical settings, irrespective of country setting and vaccination type. Although small in effect, our pooled data demonstrate favoring MPTMRs for improving vaccination uptake compared to usual care. Subanalysis by intervention characteristics, country setting, country economic status, and vaccination type did not change the effectiveness of the intervention.

Notably, substantial heterogeneity was observed in the data of the included studies; nonetheless, similar results were obtained when excluding studies with high attrition bias from the meta-analysis. The findings in Neiderhauser et al. demonstrated an effect in favor of usual care (RR = 0.65; 95%CI: 0.40; 1.06) in relation to vaccination uptake. The sample size in this study was small, and the effect observed was not statistically significant. In addition, this study reported a high loss to follow-up in the MPTMRs (39%) compared to a 10% loss to follow-up in the control arm that could have influenced the findings [49].

MPTMRs having a positive effect on vaccination uptake could be influenced by a number of factors: (1) participants in the MPTMR arm receiving an additional $20 as incentive to support cellular phone charges [58], (2) participants in the MPTMR arm receiving at least 3 MPTMRs that could have influenced adherence to scheduled vaccination appointments [44,46,65], and (3) a study conducted in a high-risk population (individuals with chronic medical condition including pregnant individuals) in Australia that could have affected the findings [45]. We also observed relatively higher effectiveness of MPTMRs in HICs than in LMICs, which could be facilitated by easier access to health care, vaccination programs, and economic advancement.

The findings of this systematic review highlight the simplicity and effectiveness of mobile phone text message reminders (MPTMRs) in improving vaccination uptake. The effectiveness of MPTMRs was not influenced by intervention characteristics, country setting, economic status, or vaccination type. This suggests that MPTMRs are a universally effective strategy across various contexts, populations, and interventions. First, intervention characteristics (whether MPTMRs were used alone or combined with other components) showed no significant modification of the intervention’s effectiveness. This indicates that the core element—reminding individuals about upcoming vaccination appointments through text messaging—remains effective regardless of additional components, such as educational content or supplementary communication methods. The simplicity of MPTMRs likely plays a key role in their wide applicability and success, as they directly address one of the most common barriers to vaccination: missed appointments due to forgetfulness. Second, our country setting subgroup analysis (urban vs. rural) also revealed no substantial differences in the effectiveness of MPTMRs. This finding is particularly important as it challenges the notion that urban areas, which often have better infrastructure and access to health services, would show a greater benefit from text message reminders. Instead, the results suggest that the intervention is equally effective in rural or semi-urban settings, where health service access is more challenging. This supports the idea that digital interventions like MPTMRs can bridge the gap in health care delivery, even in less accessible regions. Furthermore, the review did not find significant differences in effectiveness when stratifying by country economic status (low- and middle-income countries [LMICs] vs. high-income countries [HICs]). This indicates that MPTMRs have the potential to be just as effective in resource-limited settings as in more affluent ones. Although fewer studies contributed data from LMICs compared to HICs, similar results across both groups suggest that the affordability and simplicity of MPTMRs make them a feasible intervention for improving vaccination uptake, regardless of a country’s financial status. Lastly, the type of vaccination (early childhood vaccines, HPV, or seasonal influenza) did not significantly impact the effectiveness of MPTMRs. The results reflect the adaptability of MPTMRs in reminding individuals of vaccinations across different stages of life, from infancy to adulthood, and for both routine and seasonal vaccinations.

### 4.2. Comparison with the Current Literature

Our findings are in agreement with findings from other published reviews that showed that MPTMRs significantly improved childhood, adolescent, and adult vaccination coverage, including pregnant women in LMICs [28,162], irrespective of country setting [163]. A recent systematic review also showed that MPTMRs significantly improved the receipt of vaccinations (RR = 1.29; 95%CI: 1.15 to 1.44) compared to postcard reminders [26]. In addition, findings in an earlier systematic review demonstrated that weekly MPTMRs lowered the risk of non-adherence to antiretroviral therapy, although quantitative synthesis was conducted on only two RCTs with adult patients only [164].

### 4.3. Impact of Mechanisms of MPTMRs

An estimated 7.41 billion people currently are mobile phone owners with an increase to 7.49 billion people expected in 2025 [165]. Since mobile phone use has also become more widespread [166] and MPTMRs were shown to be cost-effective in distributing health information in LMICs [167], MPTMRs could be used as a tool to penetrate areas that are hard to reach, fuel adoption of health intervention and expand the reach of vaccination programs in LMICs.

### 4.4. Strengths and Limitations of Methods

This systematic review had several strengths. Specifically, we included RCTs, conducted a well-defined comprehensive search of multiple bibliographic databases, and assessed and evaluated studies irrespective of study setting, publication language, or disease. While it is true that the effect size of mobile phone text message reminders (MPTMRs) on vaccination uptake is relatively small, this study remains highly significant for several reasons. First, small effect sizes can still have a substantial impact on public health when applied at scale. Given the global decline in vaccination coverage—exacerbated by the COVID-19 pandemic—every incremental increase in vaccination uptake has the potential to prevent vaccine-preventable diseases and save lives, especially in low- and middle-income countries where vaccination rates are already suboptimal. The population-level impact of small improvements in uptake should not be underestimated, particularly when considering large-scale immunisation efforts. Secondly, the simplicity, cost-effectiveness, and broad applicability of MPTMRs make them a practical tool for health care systems worldwide. Even a modest effect is valuable, as MPTMRs require minimal resources to implement and can be easily integrated into existing health care infrastructures. This is particularly important in resource-limited settings where more complex or costly interventions may not be feasible.

Moreover, MPTMRs are a low-barrier intervention that can reach diverse populations, including those in rural or underserved areas, where access to health care and timely vaccinations is often a challenge. The lack of significant variation in the intervention’s effectiveness across different economic statuses, settings, and vaccination types further underscores its utility in a variety of contexts. Finally, this study contributes to the growing body of evidence supporting digital health interventions. It lays the groundwork for future research aimed at optimizing and combining MPTMRs with other strategies, potentially enhancing their effectiveness. The ability to deliver personalized, timely reminders with minimal effort opens avenues for further innovation in public health interventions.

These studies, therefore, represent the most recent and available published evidence that evaluated the effectiveness of MPTMRs on vaccination uptake in a wide population. In addition, we diligently applied and adhered to the international standardized guidelines for conducting and reporting systematic reviews [31]. All studies included in the review showed a low risk of bias for random sequence generation, indicating an increased likelihood that the effect observed may be attributed to MPTMRs.

### 4.5. Limitations of Included Studies

Various limitations were identified in this review that are linked to limitations of the original studies. Most studies included in this review showed an unclear risk of bias since these studies did not report information on allocation concealment. In addition, seven studies showed a high risk of attrition bias, which could have influenced the study results. We did, however, conduct sensitivity analyses to better understand the impact of these limitations.

### 4.6. Implications for Future Research

The findings of this study have shown that MPTMRs are effective irrespective of a country’s economic status and the study setting. However, the majority of studies included in this review were conducted in HICs in an urban setting, making generalizability to LMICs a challenge. In addition, these studies focused on assessing the effect of MPTMRs in a population with bigger sample sizes, possibly due to increased ownership of mobile phones in this study population, compared to LMICs in rural settings. Future research should focus on assessing the effectiveness of MPTMRs in LMICs, irrespective of vaccination type.

A recent report demonstrated that language could have a positive influence on vaccine hesitancy [168]. Therefore, tailoring MPTMRs to include language specific to the population could thus influence vaccine hesitancy, which may have a positive effect on appointment adherence and subsequently vaccination recall. More studies are needed to assess tailored MPTMRs on vaccination recall, irrespective of the population.

MPTMRs were shown to be feasible in improving physical health in individuals with psychotic disorders [169] and cost-effective in distributing information on health and reminders in LMICs [167]. Currently, limited data are available on the feasibility and cost-effectiveness of MPTMRs in routine vaccination schedules globally. Generating these data is essential in evaluating the adoptability, scalability, and sustainability of this intervention in existing vaccination programs.

### 4.7. Implications for Practice and Policy

This study may have implications for both public health practice and vaccine research. The findings of this systematic review show that MPTMRs may be a useful tool to supplement existing standard practice within the health care system to facilitate behavior changes that may promote vaccination uptake. These findings could enable evidence-based decision making and enable evaluation of critical factors that influence achieving and sustaining immunization coverage globally, irrespective of the target population, country setting, and vaccination type.

## 5. Conclusions

MPTMRs have an effect, albeit relatively small, on vaccination uptake. The intervention works broadly across various programs irrespective of the target population characteristics or nature of the intervention. Our findings indicate that MPTMRs may be an effective tool to improve vaccination uptake, irrespective of disease, country setting, and economic status. These findings may assist public health practitioners, policymakers, and vaccine researchers in evidence-based decision making that focuses on MPTMRs and their effect on vaccination coverage.

## Figures and Tables

**Table 1:** Meta-analysis, by subgroup, of the effectiveness of text message reminders for vaccination recall after enrollment visit.

Outcome or Subgroup	No of Studies	No of Participants	Pooled RR [95%CI]	I^2^ Statistic (%)	*p* Value ^a^
All Studies	25	64,536	1.09 [1.06, 1.13]	76%	-
Intervention Characteristics					0.84
Text Message PLUS Additional ^b^	13	17,394	1.10 [1.06, 1.16]	83%	
Text Message ONLY	12	47,142	1.10 [1.04, 1.15]	71%	
Country Setting					0.95
Urban	19	57,929	1.09 [1.05, 1.13]	73%	
Other ^c^	6	6607	1.10 [0.97, 1.24]	89%	
Country Economic Status					0.19
LMIC	9	7350	1.07 [1.03, 1.11]	65%	
HIC	16	57,186	1.12 [1.06, 1.18]	79%	
Vaccination Type					0.20
Early Childhood Vaccinations	16	10,480	1.07 [1.03, 1.11]	63%	
HPV	5	36,418	1.17 [1.05, 1.30]	86%	
Other ^d^	4	17,638	1.14 [1.01, 1.28]	88%	
Studies without Attrition Bias	18	55,988	1.11 [1.07, 1.15]	79%	-

RR, risk ratio; CI, confidence interval; LMIC, low- and middle-income country; HIC, high-income country; HPV, human papilloma virus. ^a^ test for subgroup differences; ^b^ additional components include interactive text messages, appointment card reminders, standard verbal counseling, educational videos, and routine health education; ^c^ includes rural, semi-urban, and suburban; ^d^ includes seasonal influenza vaccination.

## Data Availability

Detailed methods, results, and additional data are available in the manuscript and the Supplementary Materials.

## Supplementary Materials

The following supporting information can be downloaded at: https://www.mdpi.com/article/10.3390/vaccines12101151/s1, Table S1: Pre-Defined Inclusion and Exclusion Criteria for Full-Text Eligibility; Table S2: Primary Search Strategy for PubMed; Table S3: Characteristics of Excluded Studies; Table S4: Characteristics of Included Studies; Table S5: Summary of Findings. Figure S1: Summary of Risk of Bias Graph for Included Studies; Figure S2: Meta-analysis of data from included studies omitting studies of poor quality; Figure S3: Meta-analysis of data from included studies that excluded studies with high attrition bias; Figure S4: Subgroup analysis based on intervention characteristics; Figure S5: Subgroup Analysis based on country setting; Figure S6: Subgroup analysis based on country economic status; Figure S7: Subgroup analysis based on vaccination type; Figure S8: Funnel plot illustrating publication bias.
