# Supplementary material for: Mobile Phone Text Message Reminders to Improve Vaccination Uptake: A Systematic Review and Meta-Analysis
Source: Vaccines (Basel). 2024 Oct 8;12(10):1151. doi: 10.3390/vaccines12101151 (PMC11511517; doi:10.3390/vaccines12101151)
Supplement: Supplementary file 1 [file vaccines-12-01151-s001.zip › vaccines-3191635-supplementary.pdf]

Supplementary Materials to:

Mobile Phone Text Message Reminders to Improve Vaccination Uptake: A Systematic Review and Meta-Analysis

Gail Erika Louw [1,†], Ameer Steven-Jorg Hohlfeld [2,†], Robyn Kalan [1] and Mark Emmanuel Engel [1,3*]

[1] Cape Heart Institute, Department of Medicine, Faculty of Health Sciences, University of Cape Town, Cape Town 7925, South Africa; louwgail@gmail.com (G.E.L.); robyn.kalan@gmail.com (R.K.)
[2] Health Systems Research Unit, South African Medical Research Council, Tygerberg 7501, South Africa; ameer.hohlfeld@mrc.ac.za
[3] South African Cochrane Centre, South African Medical Research Council, Tygerberg 7501, South Africa
* Correspondence: mark.engel@mrc.ac.za; Tel.: +27-21-938-0307
† These authors contributed equally to this work.

Table S1: Pre-Defined Inclusion and Exclusion Criteria for Full-Text Eligibility.

	**Inclusion**	**Exclusion**
**Study design**	Randomized Control Trials	Non-Randomized Control Trials
**Intervention type**	Mobile phone text message reminders	Voice calls reminders; E-mails, Educational Videos, Autodial reminders, Postcards, Letter Correspondence
**Control type**	Usual care, including verbal communication on the next vaccination date to caregiver or participant or informing caregiver or participant about next scheduled appointment date on the immunization card	Absence of the control arm, or control arm not usual care
**Vaccination status**	Have had first dose of any vaccine in vaccination schedule	Vaccination Naïve

Table S2: Primary Search Strategy for PubMed.

**Search**	**PubMed**
#1	Immunization [MeSH Terms] OR immunis* OR immuniz* OR vaccin*
#2	adolescen* OR child* OR teenager* OR adult* OR infant* OR caregiver*
#3	“SMS” OR cellphone* OR “mobile phone*” OR “text mess*” OR “short message service*” OR “text reminder*” OR “Telegram” OR “WhatsApp” OR “social media” OR “reminder system*” OR reminder OR routine* OR “telemedicine” [MeSH Terms]
#4	(randomized controlled trial [pt] OR controlled clinical trial [pt] OR (randomized [tiab] OR placebo [tiab] OR drug therapy [sh] OR randomly [tiab] OR trial [tiab] OR groups [tiab]) NOT (animals [mh] NOT humans [mh])
#5	#1 AND #2 AND #3 AND #4

Table S3: Characteristics of Included Studies

Study	Country (Setting)	Nr of Participants	Vaccination Schedule	Intervention	Control	Outcomes
Ahlers-Schmidt (2012)	USA (Rural)	90	2-, 4-, and 6-month vaccinations	Appointment reminder text message (including appointment card at previous appointment)	Appointment Card at previous appointment	Receipt and timeliness of vaccines at 2,4- and 6-month visits
Bangure (2015)	Zimbabwe (Urban)	304	Penta-1, OPV-1, PCV-1 at 6 weeks<br>Penta-2, OPV-2, PCV-2 at 10 weeks<br>Penta-3, OPV-3, PCV-3 at 14 weeks	Appointment reminder text message (including routine health education)	Routine Health Education and informed of next appointment date	Received of vaccines at 6-, 10- and 14-week visits
Buttenheim (2022)	USA (Urban)	7479	Seasonal Influenza Vaccine (1 dose)	Appointment reminder text message and reservation of Influenza vaccine.	No text message reminder	Receipt of Influenza vaccine
Chandir (2022)	Pakistan (Urban)	3199	Penta-1, OPV-1, PCV-1 at 6 weeks<br>Penta-2, OPV-2, PCV-2 at 10 weeks<br>Penta-3, OPV-3 PCV-3 at 14 weeks<br>Measles -1 and -2 at 9 and 15 months	Appointment reminder text message	No text message reminder	Full immunization coverage at 12 months

Penta-1, 2, 3: first, second or third dose of vaccine that protects against Diptheria-Pertussis-Tetanus (DPT or DTwP) and Hepatitis B (Hep B) and diseases caused by Haemophilus Influenzae type b (Hib); **OPV-1, 2, 3**: first, second or third dose of oral polio vaccine; **PCV-1, 2, 3**: first, second or third dose of the pneumococcal conjugate vaccine

Table S3: Characteristics of Included Studies (cont.)

Study	Country (Setting)	Nr of Participants	Vaccination Schedule	Intervention	Control	Outcomes
DeCamp (2020)	USA (Urban)	157	2- dose flu vaccine and age-specific immunisation schedule from birth to 15 months	Interactive text message reminders and educational video (with Flu vaccine reminders)	No text message reminders	Up-to-date immunization up to 15 months and receipt of 2-dose flu vaccine
Dissieka (2019)[1]	Côte d'Ivoire (Rural/ Semi-urban/Urban)	1596	Penta-1 at 6 weeks,<br>Penta-2 at 10 weeks,<br>Penta-3 at 14 weeks<br>Vit A at 6 months<br>MMR and yellow fever at 9 months	Text message or voice reminders based on participant preference	No reminders	Attendance at each visit
Domek (2016)	Guatemala (Urban)	321	Penta-1, PCV-1, OPV-1, Rota-1 at 2 months<br>Penta-2, PCV-2, OPV-2, Rota-2 at 4 months<br>Penta-3, OPV-3 at 6 months	Appointment text message reminders and written reminders in immunisation card for next appointment	Immunisation card for next appointment	Completion of immunisation series
Domek (2019)	Guatemala (Rural/Urban)	720	Penta-1, PCV-1, OPV-1, Rota-1 at 2 months<br>Penta-2, PCV-2, OPV-2, Rota-2 at 4 months<br>Penta-3, OPV-3 at 6 months	Appointment text message reminders and immunisation card for next appointment	Immunisation card for next appointment	Completion and timeliness of immunisation series

[1]Study excluded from meta-analysis and risk of bias assessment since outcome data not stratified by intervention type.

Penta-1, 2, 3: first, second or third dose of vaccine that protects against Diptheria-Pertussis-Tetanus (DPT or DTwP) and Hepatitis B (Hep B) and diseases caused by Haemophilus Influenzae type b (Hib); **VitA:** vitamin A supplementation; **MMR:** vaccine that protects against Measles, Mumps and Rubella; **OPV-1, 2, 3**: first, second or third dose of oral polio vaccine; **PCV-1, 2, 3**: first, second or third dose of the pneumococcal conjugate vaccine; **Rota 1-, 2:** first or second dose of rotavirus vaccine

Table S3: Characteristics of Included Studies (cont.)

Study	Country (Setting)	Nr of Participants	Vaccination Schedule	Intervention	Control	Outcomes
Ekhaguere (2019)[1]	Nigeria (Rural)	600	Penta-1, OPV-1, PCV-1, Rota-1 at 6 weeks.<br>Penta-2, OPV-2, Rota-2, PCV-2 at 10 weeks.<br>Penta-3, OPV-3, PCV-3, IPV at 14 weeks.<br>Vit A at 6 months<br>Measles and yellow fever vaccines at 9 months	Voice call, text message and email reminders	Immunisation card for next appointment	Receipt of Penta-1, 2 and 3 vaccinations
Eze (2015)	Nigeria (Urban)	1001	DPT-1 at 6 weeks<br>DPT-2 at 10 weeks<br>DPT-3 at 14 weeks	Appointment reminder text message	No information reported	Vaccination timeliness and coverage
Gibson (2017)	Kenya (Rural)	698	Penta-1, OPV-1 at 6 weeks,<br>Penta-2, OPV-2 at 10 weeks<br>Penta-3, OPV-3 at 14 weeks,<br>Measles at 9 months	Appointment reminder text message	No information reported	Fully immunised by 12 months
Gurfinkel (2021)	USA (Urban)	24684	2-dose HPV	Appointment reminder text message	No reminders	HPV vaccination initiations and completion
Hofstetter (2015b)	USA (Urban)	1368	MMR vaccination at 13 months	Appointment reminder text message	Routine automated telephone reminders	MMR vaccination by 13 months

[1]Study excluded from meta-analysis and risk of bias assessment since outcome data not stratified by intervention type.

Penta-1-, 2, 3: first, second or third dose of vaccine that protects against Diptheria-Pertussis-Tetanus (DPT or DTwP) and Hepatitis B (Hep B) and diseases caused by Haemophilus Influenzae type b (Hib); **PCV-1, 2, 3**: first, second or third dose of the pneumococcal conjugate vaccine; **OPV-1, 2, 3**: first, second or third dose of oral polio vaccine; **Rota-1, 2:** first or second dose of rotavirus vaccine; **IPV**: Inactivated polio virus vaccine; **VitA:** vitamin A supplementation; **DPT-1, 2, 3:** first, second or third dose of vaccine against Diptheria, Pertussis and Tetanus; **HPV**: Human Papillomavirus

Table S3: Characteristics of Included Studies (cont.)

Study	Country (Setting)	Nr of Participants	Vaccination Schedule	Intervention	Control	Outcomes
Kazi (2018)	Pakistan (Urban)	300	Penta and OPV vaccines at 6, 10 and 14 weeks	Appointment reminder text message and standard verbal counseling	One standard verbal counseling at time of enrollment	Immunisation at 18 weeks
Kempe (2016)[1]	USA (Urban)	893	3-dose HPV	Preferred method of reminders (text, email or automated telephone message)	Usual care for immunization and well-care reminder or recall	Vaccination series completion rates
Lerner (2021)[1]	USA (Urban)	689	Seasonal Influenza vaccine (first and second dose)	Reminder by patient portals using text or email (based on patient portal preference)	No reminders	Completion of 2-dose Influenza vaccination
Mekonnen (2021)	Ethiopia (Urban)	434	Penta-1, OPV-1, PCV-1 and Rota-1 at 6 weeks. Penta-2, OPV-2, Rota-2 and PCV-2 at 10 weeks. Penta-3, OPV-3, PCV-3 and IPV at 14 weeks. Measles at 9 and 12 months	Appointment text message reminders and routine vaccination appointment reminders	Immunisation cards and verbal reminder of next appointment	Vaccination coverage and timeliness
Menzies (2020)	Australia (Urban)	792	Vaccines due at 2, 4, 6, 12 and 18 months	Appointment reminder text message	No reminders	Receipt of vaccines at 2, 4-, 6-, 12- and 18-months including timeliness of vaccinations

[1]Study excluded from meta-analysis and risk of bias assessment since outcome data not stratified by intervention type

Penta-1-, 2, 3: first, second or third dose of vaccine against Diptheria-Pertussis-Tetanus (DPT or DTwP) and Hepatitis B (Hep B) and diseases caused by Haemophilus Influenzae type b (Hib); **MMR:** vaccine that protects against Measles, Mumps and Rubella; **OPV-1, 2, 3**: first, second or third dose of oral polio vaccine; **HPV**: Human Papillomavirus

Table S3: Characteristics of Included Studies (cont.)

Study	Country (Setting)	Nr of Participants	Vaccination Schedule	Intervention	Control	Outcomes
Niederhauser (2015)	Hawaii (Urban)	57	DTaP, PCV, Hib, HepB and Polio at 2, 4 and 6 months	Appointment reminder text message	Age-Appropriate newborn health topics messages	Immunization compliance
O'Grady (2022)	Australia (Rural)	196	Vaccines due at 2, 4 and 6 months	Appointment reminder text message	No intervention or contact until infant turned 7 months old	Proportion of infants age-appropriately vaccinates at 7 months.
O'Leary (2015)	USA (Urban and suburban)	4587	DTaP at 11 to 12 years<br>MCV at age 11 to 12 with a booster at age 16<br>3-dose HPV vaccine series, to be started at age 11 to 12<br>Annual Influenza vaccine	Interactive, bidirectional short messages	No reminders	Completion of all needed and any vaccinations
Patel (2014)[1]	USA (Urban)	365	3-dose HPV	Appointment reminders based on preference (text message, email, phone call, private Facebook message or standard mail)	No reminders	Completion of HPV series
Rand (2015)	USA (Urban)	3812	3-dose HPV	Interactive Text message reminders	General adolescent health text messages	Receipt of first HPV dose (primary outcome), but receipt of second and third dose assessed

[1]Study excluded from meta-analysis and risk of bias assessment since outcome data not stratified by intervention type

DTaP: Vaccine against Diptheria-Tetanus and Pertussis; **PCV**: pneumococcal conjugate vaccine; **Hib:** Haemophilus Influenzae type b vaccine; **HepB:** Hepatitis B; **MCV**: Meningococcal conjugate vaccine; **HPV**: Human Papillomavirus Vaccine

Table S3: Characteristics of Included Studies (cont.)

Study	Country (Setting)	Nr of Participants randomized	Vaccination Schedule	Intervention	Control	Outcomes
Rand (2017)	USA (Urban)	391	3-dose HPV	Reminder message based on preference (text or telephone)	No reminders	Receipt of third dose of HPV vaccine and HPV vaccination rates
Regan (2017)	Australia (Urban)	12354	Seasonal Influenza Vaccine	Appointment reminder text message	No SMS reminders	Receipt of influenza vaccine
Richman (2016)[1]	USA (Rural)	264	3-dose HPV	Electronic intervention (text or e-mail appointment reminders and education messages)	Standard of care which was paper card with next appointment date	HPV vaccine completion rates
Richman (2019)[1]	USA (Rural)	257	3-dose HPV	Electronic intervention (text or e-mail appointment reminders and education messages)	Standard of care which was paper card with next appointment date	HPV vaccine completion rates
Shinde (2018)	India (Rural and semi-urban)	125	6- and 10-week vaccination schedule	Appointment reminder text message	Immunization cards of next appointment	Vaccination receipt by 10 weeks
Stockwell (2012)	USA (Urban)	Text4Health (Adolescents) = 361<br><br>Text4Health (Paediatric) = 174	Text4Health (Adolescents): MCV4 and DTaP<br><br>Text4Health (Paediatric): Hib	Text4Health (Adolescents): automated text message reminders<br><br>Text4Health (Paediatric): Text message reminders and paper mail	Text4Health (Adolescents): No immunization reminders<br><br>Text4Health (Paediatric): Paper mail	Text4Health (Adolescents): Receipt of MCV4 or Tdap<br><br>Text4Health (Paediatric): Receipt of Hib vaccine as part of primary vaccination series

[1]Study excluded from meta-analysis and risk of bias assessment since outcome data not stratified by intervention type

HPV: Human Papillomavirus Vaccine; **DTaP**: Vaccine that protects against Diptheria-Tetanus and Pertussis**; MCV**: Meningococcal conjugate vaccine; **Hib:** Haemophilus Influenzae type b vaccine

Table S3: Characteristics of Included Studies (cont.)

**Study**	**Country (Setting)**	**Nr of Participants randomized**	**Vaccination Schedule**	**Intervention**	**Control**	**Outcomes**
Stockwell (2015)	USA (Urban)	444	2-dose Influenza vaccine	Appointment reminder text message and written reminder	Written reminder only	Receipt and timeliness of second dose of influenza vaccine
Stockwell (2022)	USA (Urban)	2086	2-dose Influenza	Appointment reminder text message	Variable reminder systems*	Receipt of second dose of influenza vaccine
Tull (2019)	Australia (Urban)	2944	3-dose HPV	Self-regulatory SMS	No reminders	Receipt of any dose HPV vaccine

***** Variable reminder systems included no second dose reminders or letter, phone, email or patient portal message, or a written card
HPV: Human Papillomavirus Vaccine

Table S4: Characteristics of Excluded Studies [ordered by study ID]

Study ID	Reason for Exclusion
Adams (2012)	Ineligible outcome measured
Ahmed (2018)	Ineligible study design
Alonge (2023)	Ineligible study design
Aragones (2015)	Ineligible study design
Atchison (2013)	Ineligible study design
Atkinson (2016)	Ineligible study design
Atnafu (2017)	Ineligible outcome measured
Bar-Shain (2015)	Ineligible study design
Bay (2017)	Ineligible study design
Brigham (2012)	Ineligible intervention, not MPTMRs
Brown (2017)	Ineligible intervention, not MPTMRs
Bundy (2013)	Ineligible intervention, not MPTMRs
Bushar (2017)	Ineligible study design
Busso (2015)	Ineligible intervention, not MPTMRs
Chai (2013)	Ineligible outcome measured
Cutrona (2018)	Ineligible outcome measured
Davis (2020)	Ineligible study design
Debroy (2023)	Ineligible intervention, not MPTMRs
de Oliveira Bressane Lima (2020)	Ineligible study design
Di Mauro (2022)	Ineligible study design
Diallo (2012)	Ineligible study design
Dombkowski (2014)	Ineligible study design
Dombkowski (2017)	Ineligible study design
Erwin (2019)	Ineligible outcome measured
Fiks (2015)	Ineligible study design
Frew (2017)	Ineligible study design
Garcia-Dia (2017)	Ineligible study design
Gerend (2020)	Ineligible study design
Gerend (2021)	Ineligible outcome measured
Ghadieh (2015)	Ineligible outcome measured
Glanz (2017)	Ineligible intervention, not MPTMRs
Gold (2021)	Ineligible intervention, not MPTMRs
Haji (2016)	Ineligible study design

Table S4: Characteristics of Excluded Studies [ordered by study ID] (cont.)

Study ID	Reason for Exclusion
Haskew (2015)	Ineligible study design
Henrikson (2018)	Ineligible intervention, not MPTMRs
Herrett (2016)	Ineligible outcome measured
Hofstetter (2015)	Ineligible outcome measured
Hofstetter (2017)	Ineligible outcome measured
Hurley (2018)	Ineligible intervention, not MPTMRs
Hurley (2019)	Ineligible intervention, not MPTMRs
Ibraheem (2021)	Ineligible study design
James (2021)	Ineligible study design
Johri (2020)	Ineligible intervention, not MPTMRs
Jones Cooper (2013)	Ineligible study design
Jordan (2015)	Ineligible study design
Juon (2016)	Ineligible intervention, not MPTMRs
Kagucia (2021)	Ineligible outcome measured
Kahn (2018)	Ineligible study design
Kawakatsu (2020)	Ineligible outcome measured
Kazi (2017)	Ineligible study design
Keeshin (2017)	Ineligible study design
Kempe (2020)	Ineligible intervention, not MPTMRs
Kempe (2015)	Ineligible intervention, not MPTMRs
Kim (2017)	Ineligible study design
Kiwanuka (2018)	Ineligible outcome measured
Lee (2016)	Ineligible study design
Lee (2020)	Ineligible outcome measured
Levine (2021)	Ineligible intervention, not MPTMRs
Liao (2020)	Ineligible outcome measured
Liao (2022)	Ineligible outcome measured
Manderson (2023)	Ineligible study design
Masresha (2020)	Ineligible study design
Matheson (2014)	Ineligible study design
McGlone (2017)	Ineligible study design
Milkman (2021)	Ineligible outcome measured
Mohanty (2018)	Ineligible study design
Moniz (2013)	Ineligible outcome measured
Morris (2015)	Ineligible study design
Nehme (2019)	Ineligible outcome measured

Table S4: Characteristics of Excluded Studies [ordered by study ID] (cont.)

**Study ID**	**Reason for Exclusion**
Oladepo (2021)	Ineligible study design
O'Leary (2019)	Ineligible intervention, not MPTMRs
Patel (2023)	Ineligible outcome measured
Qamar (2020)	Ineligible study design
Regan (2023)	Ineligible study design
Schlumberger (2015)	Ineligible study design
Seth (2018)	Ineligible outcome measured
Staras (2021)	Ineligible outcome measured
Stockwell (2012)	Ineligible outcome measured
Stockwell (2014)	Ineligible outcome measured
Suh (2012)	Ineligible intervention, not MPTMRs
Suppli (2017)	Ineligible study design
Szilagyi (2013)	Ineligible intervention, not MPTMRs
Szilagyi (2019)	Ineligible outcome measured
Szilagyi (2020a)	Ineligible intervention, not MPTMRs
Szilagyi (2020b)	Ineligible outcome measured
Szilagyi (2022)	Ineligible intervention, not MPTMRs
Tuckerman (2023)	Ineligible outcome measured
Ueberroth (2022)	Ineligible outcome measured
Venci (2015)	Ineligible study design
Venkatesh (2020)	Ineligible study design
Wagner (2021)	Ineligible outcome measured
Wijesundara (2020)	Ineligible outcome measured
Wynn (2021)	Ineligible comparison
Xeuatvongsa (2016)	Ineligible study design
Yeung (2018)	Ineligible outcome measured
Yudin (2017)	Ineligible outcome measured
Yunusa (2022)	Ineligible study design

Table S5: Summary of Findings

Messaging services compared to standard of care for Vaccination recall

Patient or population: Vaccination recall
Setting: Primary setting
Intervention: MPTMRs
Comparison: standard of care

Outcomes	Anticipated absolute effects* (95% CI)		Relative effect (95% CI)	№ of participants (studies)	Certainty of the evidence (GRADE)	Comments
	Risk with standard of care	Risk with messaging services				
Vaccination recall (Pooled data)	285 per 1,000	311 per 1,000 (302 to 322)	**RR 1.09** (1.06 to 1.13)	64536 (25 RCTs)	⊕⊕◯◯ Low[a,b]	The evidence suggests messaging services results in a slight increase in vaccination recall.
MPTMRs with additional components	411 per 1,000	453 per 1,000 (428 to 477)	**RR 1.10** (1.04 to 1.16)	17394 (13 RCTs)	⊕⊕◯◯ Low[a,b]	The evidence suggests messaging services results in a slight increase in mPTMRs (+).
MPTMRs (alone)	238 per 1,000	260 per 1,000 (248 to 274)	**RR 1.09** (1.04 to 1.15)	47142 (12 RCTs)	⊕⊕◯◯ Low[a,b]	The evidence suggests messaging services results in a slight increase in mPTMRs (alone).
Country setting (Urban)	278 per 1,000	306 per 1,000 (295 to 317)	**RR 1.10** (1.06 to 1.14)	57929 (19 RCTs)	⊕⊕◯◯ Low[a,b]	The evidence suggests messaging services results in a slight increase in country setting (Urban).
Country setting (Rural and/or suburban and/or semi-urban)	348 per 1,000	383 per 1,000 (338 to 432)	**RR 1.10** (0.97 to 1.24)	6607 (6 RCTs)	⊕◯◯◯ Very low[c,d]	The evidence is very uncertain about the effect of messaging services on country setting (Rural).
LMIC	671 per 1,000	718 per 1,000 (691 to 745)	**RR 1.07** (1.03 to 1.11)	7350 (9 RCTs)	⊕⊕◯◯ Low[a,b]	The evidence suggests messaging services results in a slight increase in LMIC.
HIC	236 per 1,000	264 per 1,000 (250 to 278)	**RR 1.12** (1.06 to 1.18)	57186 (16 RCTs)	⊕⊕◯◯ Low[a,b]	The evidence suggests messaging services results in a slight increase in HIC.
Early Childhood	615 per 1,000	658 per 1,000 (633 to 682)	**RR 1.07** (1.03 to 1.11)	10480 (16 RCTs)	⊕◯◯◯ Very low[a,b,d,e]	The evidence is very uncertain about the effect of messaging services on early Childhood.
HPV	215 per 1,000	252 per 1,000 (226 to 280)	**RR 1.17** (1.05 to 1.30)	36418 (5 RCTs)	⊕◯◯◯ Very low[b,f,g]	The evidence is very uncertain about the effect of messaging services on HPV.

Messaging services compared to standard of care for Vaccination recall

Patient or population: Vaccination recall
Setting: Primary setting
Intervention: MPTMRs
Comparison: standard of care

Outcomes	Anticipated absolute effects* (95% CI)		Relative effect (95% CI)	№ of participants (studies)	Certainty of the evidence (GRADE)	Comments
	Risk with standard of care	Risk with messaging services				
Seasonal Influenza	236 per 1,000	269 per 1,000 (239 to 302)	**RR 1.14** (1.01 to 1.28)	17638 (4 RCTs)	⨁◯◯◯ Very low[c,f,h]	The evidence is very uncertain about the effect of messaging services on seasonal Influenza.
Omitting poor quality studies	274 per 1,000	305 per 1,000 (294 to 315)	**RR 1.11** (1.07 to 1.15)	55988 (19 RCTs)	⨁⨁⨁⨁ High[i]	Messaging services results in a slight increase in omitting poor quality.
Omitting High Attrition Bias Studies	274 per 1,000	305 per 1,000 (294 to 315)	**RR 1.11** (1.07 to 1.15)	55988 (18 RCTs)	⨁⨁⨁◯ Moderate[b,j]	Messaging services probably results in a slight increase in omitting High Attrition Bias Studies.

***The risk in the intervention group** (and its 95% confidence interval) is based on the assumed risk in the comparison group and the **relative effect** of the intervention (and its 95% CI).

CI: confidence interval; **RR:** risk ratio

GRADE Working Group grades of evidence
High certainty: we are very confident that the true effect lies close to that of the estimate of the effect.
Moderate certainty: we are moderately confident in the effect estimate: the true effect is likely to be close to the estimate of the effect, but there is a possibility that it is substantially different.
Low certainty: our confidence in the effect estimate is limited: the true effect may be substantially different from the estimate of the effect.
Very low certainty: we have very little confidence in the effect estimate: the true effect is likely to be substantially different from the estimate of effect.

Explanations

a. -1 (Mixed quality across studies)
b. -1 (High heterogeneity)
c. -2 (Very high heterogeneity)
d. -1 (Large imprecision, wide confidence interval that crosses 1)
e. The CI (1.03 to 1.11) is narrow. Assuming a threshold for small effect at 1% or 10 per 1,000, this CI may cross one threshold
f. -2 (Large imprecision)
g. The CI (1.05 to 1.30) likely crosses two thresholds (small and moderate effects)
h. The CI (1.01 to 1.28) likely crosses two thresholds (small and moderate effects)
i. Large sample size (23172 vs 23283) Narrow confidence interval (1.03 to 1.07) that doesn't cross 1 Assessment: No serious concerns if we assume the threshold for a small effect is outside this range. If the threshold for a small effect is within this range, we might consider rating down by one level
j. Large sample size (27884 vs 28104) Confidence interval (1.07 to 1.15) doesn't cross 1 Assessment: No serious concerns if we assume the threshold for a small effect is outside this range.

Certainty assessment							№ of patients		Effect		Certainty	Importance
№ of studies	Study design	Risk of bias	Inconsistency	Indirectness	Imprecision	Other considerations	messaging services	standard of care	Relative (95% CI)	Absolute (95% CI)		
**Vaccination recall (Pooled data)**												
25	randomised trials	serious[a]	serious[b]	not serious	not serious	none	9918/32159 (30.8%)	9237/32377 (28.5%)	**RR 1.09** (1.06 to 1.13)	**26 more per 1,000** (from 17 more to 37 more)	⨁⨁◯◯ Low[a,b]	
**MPTMRs with additional components**												
13	randomised trials	serious[a]	serious[b]	not serious	not serious	none	3866/8588 (45.0%)	3623/8806 (41.1%)	**RR 1.10** (1.04 to 1.16)	**41 more per 1,000** (from 16 more to 66 more)	⨁⨁◯◯ Low[a,b]	
**MPTMRs (alone)**												
12	randomised trials	serious[a]	serious[b]	not serious	not serious	none	6052/23571 (25.7%)	5614/23571 (23.8%)	**RR 1.09** (1.04 to 1.15)	**21 more per 1,000** (from 10 more to 36 more)	⨁⨁◯◯ Low[a,b]	
**Country setting (Urban)**												
19	randomised trials	serious[a]	serious[b]	not serious	not serious	none	8600/28919 (29.7%)	8064/29010 (27.8%)	**RR 1.10** (1.06 to 1.14)	**28 more per 1,000** (from 17 more to 39 more)	⨁⨁◯◯ Low[a,b]	
**Country setting (Rural and/or suburban and/or semi-urban)**												
6	randomised trials	not serious	very serious[c]	not serious	serious[d]	none	1318/3240 (40.7%)	1173/3367 (34.8%)	**RR 1.10** (0.97 to 1.24)	**35 more per 1,000** (from 10 fewer to 84 more)	⨁◯◯◯ Very low[c,d]	
**LMIC**												
9	randomised trials	serious[a]	serious[b]	not serious	not serious	none	2635/3673 (71.7%)	2467/3677 (67.1%)	**RR 1.07** (1.03 to 1.11)	**47 more per 1,000** (from 20 more to 74 more)	⨁⨁◯◯ Low[a,b]	
**HIC**												
16	randomised trials	serious[a]	serious[b]	not serious	not serious	none	7283/28486 (25.6%)	6770/28700 (23.6%)	**RR 1.12** (1.06 to 1.18)	**28 more per 1,000** (from 14 more to 42 more)	⨁⨁◯◯ Low[a,b]	
**Early Childhood**												
16	randomised trials	serious[a]	serious[b]	not serious	serious[d,e]	none	3430/5262 (65.2%)	3207/5218 (61.5%)	**RR 1.07** (1.03 to 1.11)	**43 more per 1,000** (from 18 more to 68 more)	⨁◯◯◯ Very low[a,b,d,e]	
**HPV**												

Certainty assessment							№ of patients		Effect		Certainty	Importance
№ of studies	Study design	Risk of bias	Inconsistency	Indirectness	Imprecision	Other considerations	messaging services	standard of care	Relative (95% CI)	Absolute (95% CI)		
5	randomised trials	not serious	serious[b]	not serious	very serious[f,g]	none	4155/18071 (23.0%)	3948/18347 (21.5%)	**RR 1.17** (1.05 to 1.30)	**37 more per 1,000** (from 11 more to 65 more)	⊕◯◯◯ Very low[b,f,g]	
**Seasonal Influenza**												
4	randomised trials	not serious	very serious[c]	not serious	very serious[f,h]	none	2333/8826 (26.4%)	2082/8812 (23.6%)	**RR 1.14** (1.01 to 1.28)	**33 more per 1,000** (from 2 more to 66 more)	⊕◯◯◯ Very low[c,f,h]	
**Omitting poor quality studies**												
19	randomised trials	not serious	not serious	not serious	not serious[i]	none	8271/27884 (29.7%)	7710/28104 (27.4%)	**RR 1.11** (1.07 to 1.15)	**30 more per 1,000** (from 19 more to 41 more)	⊕⊕⊕⊕ High[i]	
**Omitting High Attrition Bias Studies**												
18	randomised trials	not serious	serious[b]	not serious	not serious[j]	none	8271/27884 (29.7%)	7710/28104 (27.4%)	**RR 1.11** (1.07 to 1.15)	**30 more per 1,000** (from 19 more to 41 more)	⊕⊕⊕◯ Moderate[b,j]	

CI: confidence interval; **RR:** risk ratio

Explanations

a. -1 (Mixed quality across studies)
b. -1 (High heterogeneity)
c. -2 (Very high heterogeneity)
d. -1 (Large imprecision, wide confidence interval that crosses 1)
e. The CI (1.03 to 1.11) is narrow. Assuming a threshold for small effect at 1% or 10 per 1,000, this CI may cross one threshold
f. -2 (Large imprecision)
g. The CI (1.05 to 1.30) likely crosses two thresholds (small and moderate effects)
h. The CI (1.01 to 1.28) likely crosses two thresholds (small and moderate effects)
i. Large sample size (23172 vs 23283) Narrow confidence interval (1.03 to 1.07) that doesn't cross 1 Assessment: No serious concerns if we assume the threshold for a small effect is outside this range. If the threshold for a small effect is within this range, we might consider rating down by one level
j. Large sample size (27884 vs 28104) Confidence interval (1.07 to 1.15) doesn't cross 1 Assessment: No serious concerns if we assume the threshold for a small effect is outside this range.

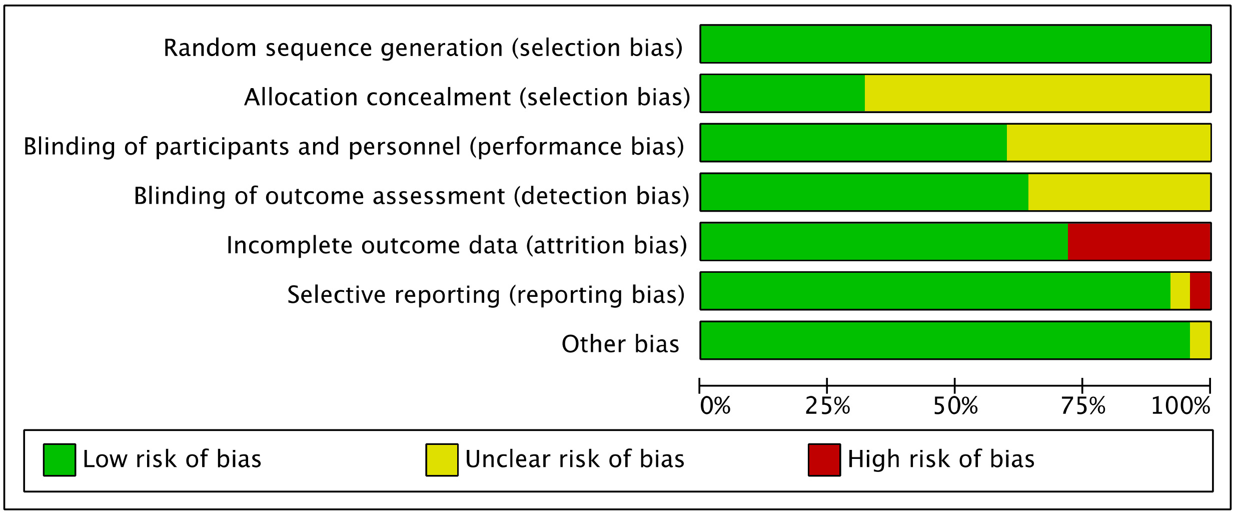

Figure S1: Summary of Risk of Bias Graph for Included Studies.

Study or Subgroup	Text Message Reminder Events	Text Message Reminder Total	Usual Care Events	Usual Care Total	Weight	Risk Ratio M-H, Random, 95% CI	Year
Ahlers-Schmidt 2012	45	50	36	40	1.8%	1.00 [0.87, 1.15]	2012
Rand 2015	141	1893	121	1919	0.7%	1.18 [0.93, 1.49]	2015
Stockwell 2015	150	225	125	219	1.6%	1.17 [1.01, 1.35]	2015
Bangure 2015	146	152	122	152	4.1%	1.20 [1.10, 1.30]	2015
Hofstetter 2015b	418	686	417	682	4.1%	1.00 [0.92, 1.08]	2015
Eze 2015	312	500	273	501	2.9%	1.15 [1.03, 1.27]	2015
Domek 2016	152	160	145	161	6.5%	1.05 [0.99, 1.12]	2016
Gibson 2017	387	476	359	489	5.7%	1.11 [1.03, 1.19]	2017
Shinde 2018	50	64	38	50	0.9%	1.03 [0.84, 1.26]	2018
Kazi 2018	114	150	107	150	1.9%	1.07 [0.93, 1.22]	2018
Tull 2019	1262	1418	1300	1526	14.0%	1.04 [1.02, 1.07]	2019
Domek 2019	330	358	333	362	10.0%	1.00 [0.96, 1.05]	2019
Menzies 2020	151	398	147	394	1.1%	1.02 [0.85, 1.22]	2020
Gurfinkel 2021	2209	12341	2111	12343	7.8%	1.05 [0.99, 1.10]	2021
Mekonnen 2021	210	213	203	213	12.3%	1.03 [1.00, 1.07]	2021
Stockwell 2022	875	1044	843	1042	10.8%	1.04 [1.00, 1.08]	2022
O'Grady 2022	57	64	53	67	1.5%	1.13 [0.97, 1.31]	2022
Chandir 2022	934	1600	887	1599	6.8%	1.05 [0.99, 1.12]	2022
Buttenheim 2022	756	1380	708	1374	5.5%	1.06 [0.99, 1.14]	2022
**Total (95% CI)**		**23172**		**23283**	**100.0%**	**1.05 [1.03, 1.07]**	
Total events	8699		8328				

Heterogeneity: $Tau^2 = 0.00$; $Chi^2 = 26.82$, df = 18 (P = 0.08); $I^2 = 33\%$
Test for overall effect: Z = 5.27 (P < 0.00001)

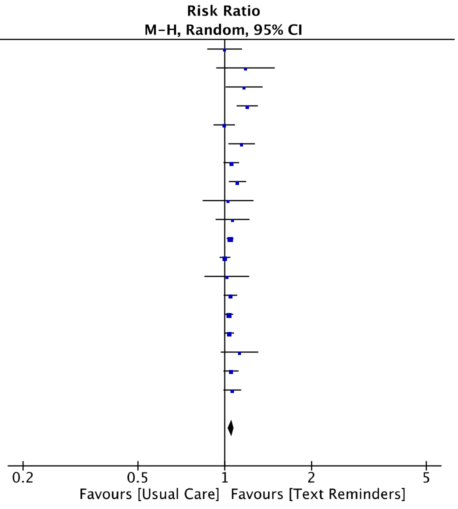

Figure S2: Meta-analysis of data from included studies omitting studies of poor quality

Study or Subgroup	Text Message Reminder Events	Text Message Reminder Total	Usual Care Events	Usual Care Total	Weight	Risk Ratio M-H, Random, 95% CI	Year
Stockwell 2012	47	282	17	253	0.5%	2.48 [1.46, 4.21]	2012
Stockwell 2015	150	225	125	219	4.0%	1.17 [1.01, 1.35]	2015
Bangure 2015	146	152	122	152	6.5%	1.20 [1.10, 1.30]	2015
Hofstetter 2015b	418	686	417	682	6.5%	1.00 [0.92, 1.08]	2015
O'Leary 2015	449	2228	354	2359	4.7%	1.34 [1.18, 1.52]	2015
Domek 2016	152	160	145	161	7.5%	1.05 [0.99, 1.12]	2016
Rand 2017	94	191	62	200	1.9%	1.59 [1.23, 2.04]	2017
Regan 2017	552	6177	406	6177	4.9%	1.36 [1.20, 1.54]	2017
Gibson 2017	387	476	359	489	7.3%	1.11 [1.03, 1.19]	2017
Kazi 2018	114	150	107	150	4.4%	1.07 [0.93, 1.22]	2018
Tull 2019	1262	1418	1300	1526	8.9%	1.04 [1.02, 1.07]	2019
Menzies 2020	151	398	147	394	3.2%	1.02 [0.85, 1.22]	2020
DeCamp 2020	64	79	52	78	3.0%	1.22 [1.01, 1.47]	2020
Mekonnen 2021	210	213	203	213	8.7%	1.03 [1.00, 1.07]	2021
Gurfinkel 2021	2209	12341	2111	12343	7.9%	1.05 [0.99, 1.10]	2021
O'Grady 2022	57	64	53	67	4.0%	1.13 [0.97, 1.31]	2022
Chandir 2022	934	1600	887	1599	7.6%	1.05 [0.99, 1.12]	2022
Stockwell 2022	875	1044	843	1042	8.5%	1.04 [1.00, 1.08]	2022
**Total (95% CI)**		**27884**		**28104**	**100.0%**	**1.11 [1.07, 1.15]**	
Total events	8271		7710				

Heterogeneity: $Tau^2 = 0.00$; $Chi^2 = 80.88$, df = 17 (P < 0.00001); $I^2 = 79\%$
Test for overall effect: Z = 5.11 (P < 0.00001)

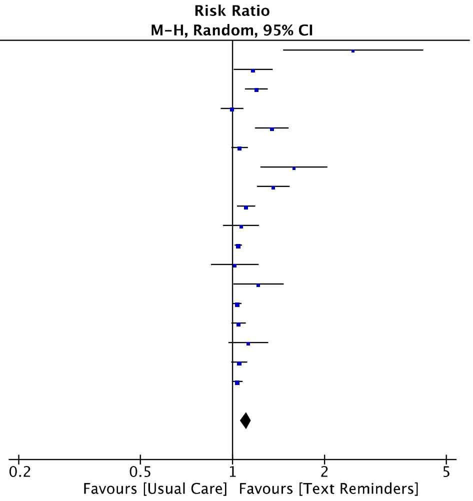

Figure S3: Meta-analysis of data from included studies that excluded studies with high attrition bias.

Study or Subgroup	Text Message Reminder Events	Total	Usual Care Events	Total	Weight	Risk Ratio M-H, Random, 95% CI	Year
**2.1.1 Text message PLUS additional**							
Stockwell 2012	47	282	17	253	0.4%	2.48 [1.46, 4.21]	2012
Ahlers-Schmidt 2012	45	50	36	40	3.3%	1.00 [0.87, 1.15]	2012
Rand 2015	141	1893	121	1919	1.6%	1.18 [0.93, 1.49]	2015
Stockwell 2015	150	225	125	219	3.0%	1.17 [1.01, 1.35]	2015
Bangure 2015	146	152	122	152	4.9%	1.20 [1.10, 1.30]	2015
O'Leary 2015	449	2228	354	2359	3.6%	1.34 [1.18, 1.52]	2015
Domek 2016	152	160	145	161	5.8%	1.05 [0.99, 1.12]	2016
Kazi 2018	114	150	107	150	3.3%	1.07 [0.93, 1.22]	2018
Tull 2019	1262	1418	1300	1526	6.9%	1.04 [1.02, 1.07]	2019
Domek 2019	330	358	333	362	6.5%	1.00 [0.96, 1.05]	2019
DeCamp 2020	64	79	52	78	2.2%	1.22 [1.01, 1.47]	2020
Mekonnen 2021	210	213	203	213	6.8%	1.03 [1.00, 1.07]	2021
Buttenheim 2022	756	1380	708	1374	5.5%	1.06 [0.99, 1.14]	2022
**Subtotal (95% CI)**		**8588**		**8806**	**53.8%**	**1.10 [1.04, 1.16]**	
Total events	3866		3623				
Heterogeneity: Tau² = 0.01; Chi² = 69.85, df = 12 (P < 0.00001); I² = 83%							
Test for overall effect: Z = 3.64 (P = 0.0003)							
**2.1.2 Text message ONLY**							
Hofstetter 2015b	418	686	417	682	5.0%	1.00 [0.92, 1.08]	2015
Niederhauser 2015	13	30	18	27	0.4%	0.65 [0.40, 1.06]	2015
Eze 2015	312	500	273	501	4.2%	1.15 [1.03, 1.27]	2015
Gibson 2017	387	476	359	489	5.6%	1.11 [1.03, 1.19]	2017
Rand 2017	94	191	62	200	1.4%	1.59 [1.23, 2.04]	2017
Regan 2017	552	6177	406	6177	3.7%	1.36 [1.20, 1.54]	2017
Shinde 2018	50	64	38	50	2.0%	1.03 [0.84, 1.26]	2018
Menzies 2020	151	398	147	394	2.4%	1.02 [0.85, 1.22]	2020
Gurfinkel 2021	2209	12341	2111	12343	6.1%	1.05 [0.99, 1.10]	2021
O'Grady 2022	57	64	53	67	3.0%	1.13 [0.97, 1.31]	2022
Chandir 2022	934	1600	887	1599	5.9%	1.05 [0.99, 1.12]	2022
Stockwell 2022	875	1044	843	1042	6.6%	1.04 [1.00, 1.08]	2022
**Subtotal (95% CI)**		**23571**		**23571**	**46.2%**	**1.09 [1.04, 1.15]**	
Total events	6052		5614				
Heterogeneity: Tau² = 0.00; Chi² = 38.49, df = 11 (P < 0.0001); I² = 71%							
Test for overall effect: Z = 3.29 (P = 0.001)							
**Total (95% CI)**		**32159**		**32377**	**100.0%**	**1.09 [1.06, 1.13]**	
Total events	9918		9237				
Heterogeneity: Tau² = 0.00; Chi² = 98.93, df = 24 (P < 0.00001); I² = 76%							
Test for overall effect: Z = 5.15 (P < 0.00001)							
Test for subgroup differences: Chi² = 0.04, df = 1 (P = 0.84), I² = 0%							

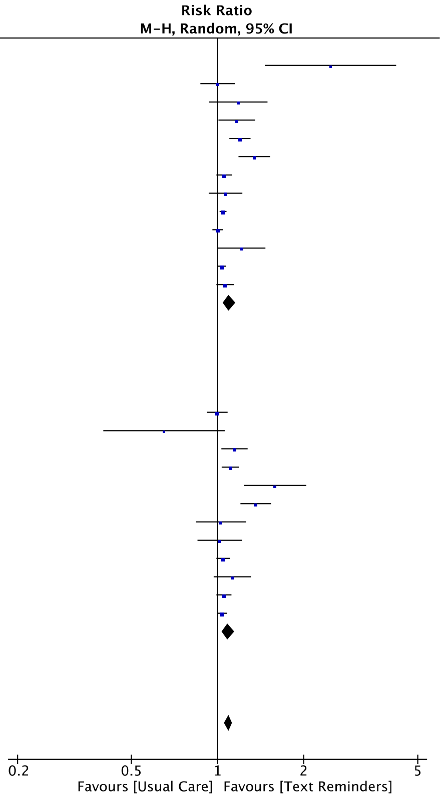

Figure S4: Subgroup analysis based on intervention characteristics.

Study or Subgroup	Text Message Reminder Events	Total	Usual Care Events	Total	Weight	Risk Ratio M-H, Random, 95% CI	Year
**3.1.1 Urban**							
Stockwell 2012	47	282	17	253	0.4%	2.48 [1.46, 4.21]	2012
Rand 2015	141	1893	121	1919	1.6%	1.18 [0.93, 1.49]	2015
Stockwell 2015	150	225	125	219	3.0%	1.17 [1.01, 1.35]	2015
Bangure 2015	146	152	122	152	4.9%	1.20 [1.10, 1.30]	2015
Eze 2015	312	500	273	501	4.2%	1.15 [1.03, 1.27]	2015
Hofstetter 2015b	418	686	417	682	5.0%	1.00 [0.92, 1.08]	2015
Niederhauser 2015	13	30	18	27	0.4%	0.65 [0.40, 1.06]	2015
Domek 2016	152	160	145	161	5.8%	1.05 [0.99, 1.12]	2016
Rand 2017	94	191	62	200	1.4%	1.59 [1.23, 2.04]	2017
Regan 2017	552	6177	406	6177	3.7%	1.36 [1.20, 1.54]	2017
Kazi 2018	114	150	107	150	3.3%	1.07 [0.93, 1.22]	2018
Tull 2019	1262	1418	1300	1526	6.9%	1.04 [1.02, 1.07]	2019
DeCamp 2020	64	79	52	78	2.2%	1.22 [1.01, 1.47]	2020
Menzies 2020	151	398	147	394	2.4%	1.02 [0.85, 1.22]	2020
Mekonnen 2021	210	213	203	213	6.8%	1.03 [1.00, 1.07]	2021
Gurfinkel 2021	2209	12341	2111	12343	6.1%	1.05 [0.99, 1.10]	2021
Stockwell 2022	875	1044	843	1042	6.6%	1.04 [1.00, 1.08]	2022
Chandir 2022	934	1600	887	1599	5.9%	1.05 [0.99, 1.12]	2022
Buttenheim 2022	756	1380	708	1374	5.5%	1.06 [0.99, 1.14]	2022
**Subtotal (95% CI)**		**28919**		**29010**	**76.2%**	**1.09 [1.05, 1.13]**	
Total events	8600		8064				
Heterogeneity: Tau² = 0.00; Chi² = 66.66, df = 18 (P < 0.00001); I² = 73%							
Test for overall effect: Z = 4.73 (P < 0.00001)							
**3.1.2 Other**							
Ahlers-Schmidt 2012	45	50	36	40	3.3%	1.00 [0.87, 1.15]	2012
O'Leary 2015	449	2228	354	2359	3.6%	1.34 [1.18, 1.52]	2015
Gibson 2017	387	476	359	489	5.6%	1.11 [1.03, 1.19]	2017
Shinde 2018	50	64	38	50	2.0%	1.03 [0.84, 1.26]	2018
Domek 2019	330	358	333	362	6.5%	1.00 [0.96, 1.05]	2019
O'Grady 2022	57	64	53	67	3.0%	1.13 [0.97, 1.31]	2022
**Subtotal (95% CI)**		**3240**		**3367**	**23.8%**	**1.10 [0.97, 1.24]**	
Total events	1318		1173				
Heterogeneity: Tau² = 0.02; Chi² = 47.05, df = 5 (P < 0.00001); I² = 89%							
Test for overall effect: Z = 1.45 (P = 0.15)							
**Total (95% CI)**		**32159**		**32377**	**100.0%**	**1.09 [1.06, 1.13]**	
Total events	9918		9237				
Heterogeneity: Tau² = 0.00; Chi² = 98.93, df = 24 (P < 0.00001); I² = 76%							
Test for overall effect: Z = 5.15 (P < 0.00001)							
Test for subgroup differences: Chi² = 0.00, df = 1 (P = 0.95), I² = 0%							

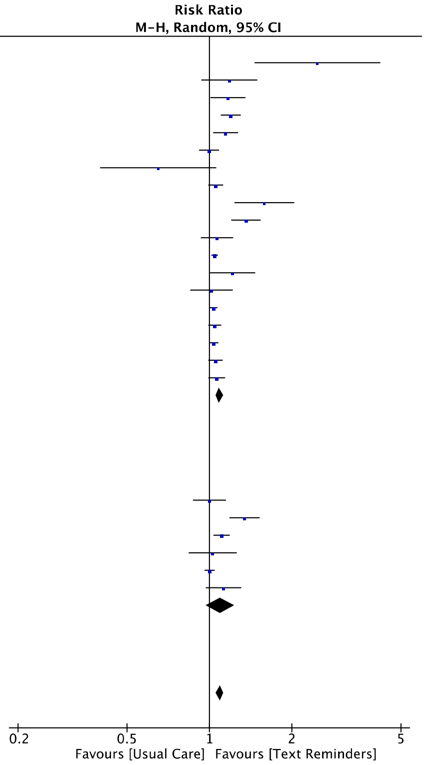

Figure S5: Subgroup analysis based on country setting

Study or Subgroup	Text Message Reminder Events	Text Message Reminder Total	Usual Care Events	Usual Care Total	Weight	Risk Ratio M-H, Random, 95% CI	Year
**4.1.1 LMIC**							
Bangure 2015	146	152	122	152	4.9%	1.20 [1.10, 1.30]	2015
Eze 2015	312	500	273	501	4.2%	1.15 [1.03, 1.27]	2015
Domek 2016	152	160	145	161	5.8%	1.05 [0.99, 1.12]	2016
Gibson 2017	387	476	359	489	5.6%	1.11 [1.03, 1.19]	2017
Shinde 2018	50	64	38	50	2.0%	1.03 [0.84, 1.26]	2018
Kazi 2018	114	150	107	150	3.3%	1.07 [0.93, 1.22]	2018
Domek 2019	330	358	333	362	6.5%	1.00 [0.96, 1.05]	2019
Mekonnen 2021	210	213	203	213	6.8%	1.03 [1.00, 1.07]	2021
Chandir 2022	934	1600	887	1599	5.9%	1.05 [0.99, 1.12]	2022
**Subtotal (95% CI)**		**3673**		**3677**	**45.0%**	**1.07 [1.03, 1.11]**	
Total events	2635		2467				
Heterogeneity: Tau^2 = 0.00; Chi^2 = 22.60, df = 8 (P = 0.004); I^2 = 65%							
Test for overall effect: Z = 3.37 (P = 0.0007)							
**4.1.2 High Income**							
Ahlers-Schmidt 2012	45	50	36	40	3.3%	1.00 [0.87, 1.15]	2012
Stockwell 2012	47	282	17	253	0.4%	2.48 [1.46, 4.21]	2012
O'Leary 2015	449	2228	354	2359	3.6%	1.34 [1.18, 1.52]	2015
Hofstetter 2015b	418	686	417	682	5.0%	1.00 [0.92, 1.08]	2015
Niederhauser 2015	13	30	18	27	0.4%	0.65 [0.40, 1.06]	2015
Rand 2015	141	1893	121	1919	1.6%	1.18 [0.93, 1.49]	2015
Stockwell 2015	150	225	125	219	3.0%	1.17 [1.01, 1.35]	2015
Rand 2017	94	191	62	200	1.4%	1.59 [1.23, 2.04]	2017
Regan 2017	552	6177	406	6177	3.7%	1.36 [1.20, 1.54]	2017
Tull 2019	1262	1418	1300	1526	6.9%	1.04 [1.02, 1.07]	2019
DeCamp 2020	64	79	52	78	2.2%	1.22 [1.01, 1.47]	2020
Menzies 2020	151	398	147	394	2.4%	1.02 [0.85, 1.22]	2020
Gurfinkel 2021	2209	12341	2111	12343	6.1%	1.05 [0.99, 1.10]	2021
Buttenheim 2022	756	1380	708	1374	5.5%	1.06 [0.99, 1.14]	2022
Stockwell 2022	875	1044	843	1042	6.6%	1.04 [1.00, 1.08]	2022
O'Grady 2022	57	64	53	67	3.0%	1.13 [0.97, 1.31]	2022
**Subtotal (95% CI)**		**28486**		**28700**	**55.0%**	**1.12 [1.06, 1.18]**	
Total events	7283		6770				
Heterogeneity: Tau^2 = 0.01; Chi^2 = 72.45, df = 15 (P < 0.00001); I^2 = 79%							
Test for overall effect: Z = 4.06 (P < 0.0001)							
**Total (95% CI)**		**32159**		**32377**	**100.0%**	**1.09 [1.06, 1.13]**	
Total events	9918		9237				
Heterogeneity: Tau^2 = 0.00; Chi^2 = 98.93, df = 24 (P < 0.00001); I^2 = 76%							
Test for overall effect: Z = 5.15 (P < 0.00001)							
Test for subgroup differences: Chi^2 = 1.73, df = 1 (P = 0.19), I^2 = 42.3%							

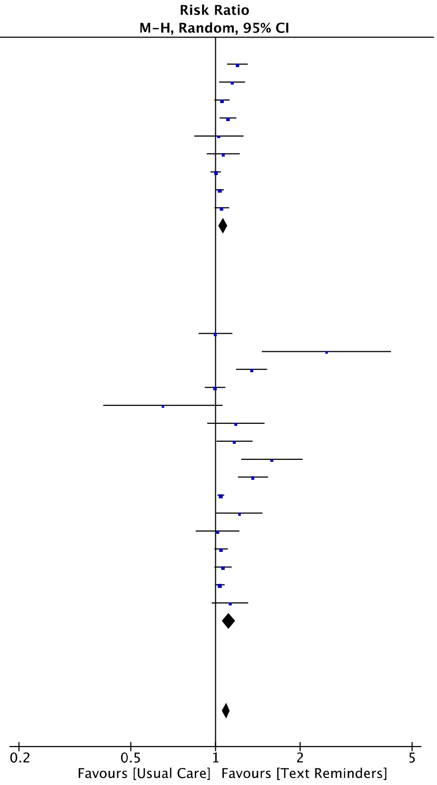

Figure S6: Subgroup analysis based on country economic status

Study or Subgroup	Text Message Reminder Events	Text Message Reminder Total	Usual Care Events	Usual Care Total	Weight	Risk Ratio M-H, Random, 95% CI	Year
**5.1.1 Early Childhood Vaccinations**							
Stockwell 2012	47	282	17	253	0.4%	2.48 [1.46, 4.21]	2012
Ahlers-Schmidt 2012	45	50	36	40	3.3%	1.00 [0.87, 1.15]	2012
Bangure 2015	146	152	122	152	4.9%	1.20 [1.10, 1.30]	2015
Eze 2015	312	500	273	501	4.2%	1.15 [1.03, 1.27]	2015
Hofstetter 2015b	418	686	417	682	5.0%	1.00 [0.92, 1.08]	2015
Niederhauser 2015	13	30	18	27	0.4%	0.65 [0.40, 1.06]	2015
Domek 2016	152	160	145	161	5.8%	1.05 [0.99, 1.12]	2016
Gibson 2017	387	476	359	489	5.6%	1.11 [1.03, 1.19]	2017
Shinde 2018	50	64	38	50	2.0%	1.03 [0.84, 1.26]	2018
Kazi 2018	114	150	107	150	3.3%	1.07 [0.93, 1.22]	2018
Domek 2019	330	358	333	362	6.5%	1.00 [0.96, 1.05]	2019
DeCamp 2020	64	79	52	78	2.2%	1.22 [1.01, 1.47]	2020
Menzies 2020	151	398	147	394	2.4%	1.02 [0.85, 1.22]	2020
Mekonnen 2021	210	213	203	213	6.8%	1.03 [1.00, 1.07]	2021
Chandir 2022	934	1600	887	1599	5.9%	1.05 [0.99, 1.12]	2022
O'Grady 2022	57	64	53	67	3.0%	1.13 [0.97, 1.31]	2022
**Subtotal (95% CI)**		**5262**		**5218**	**61.6%**	**1.07 [1.03, 1.11]**	
Total events	3430		3207				
Heterogeneity: Tau^2 = 0.00; Chi^2 = 40.98, df = 15 (P = 0.0003); I^2 = 63%							
Test for overall effect: Z = 3.32 (P = 0.0009)							
**5.1.2 HPV**							
O'Leary 2015	449	2228	354	2359	3.6%	1.34 [1.18, 1.52]	2015
Rand 2015	141	1893	121	1919	1.6%	1.18 [0.93, 1.49]	2015
Rand 2017	94	191	62	200	1.4%	1.59 [1.23, 2.04]	2017
Tull 2019	1262	1418	1300	1526	6.9%	1.04 [1.02, 1.07]	2019
Gurfinkel 2021	2209	12341	2111	12343	6.1%	1.05 [0.99, 1.10]	2021
**Subtotal (95% CI)**		**18071**		**18347**	**19.6%**	**1.17 [1.05, 1.30]**	
Total events	4155		3948				
Heterogeneity: Tau^2 = 0.01; Chi^2 = 28.91, df = 4 (P < 0.00001); I^2 = 86%							
Test for overall effect: Z = 2.95 (P = 0.003)							
**5.1.3 Other (eg. Seasonal Influenza Vaccination)**							
Stockwell 2015	150	225	125	219	3.0%	1.17 [1.01, 1.35]	2015
Regan 2017	552	6177	406	6177	3.7%	1.36 [1.20, 1.54]	2017
Stockwell 2022	875	1044	843	1042	6.6%	1.04 [1.00, 1.08]	2022
Buttenheim 2022	756	1380	708	1374	5.5%	1.06 [0.99, 1.14]	2022
**Subtotal (95% CI)**		**8826**		**8812**	**18.8%**	**1.14 [1.01, 1.28]**	
Total events	2333		2082				
Heterogeneity: Tau^2 = 0.01; Chi^2 = 25.59, df = 3 (P < 0.0001); I^2 = 88%							
Test for overall effect: Z = 2.12 (P = 0.03)							
**Total (95% CI)**		**32159**		**32377**	**100.0%**	**1.09 [1.06, 1.13]**	
Total events	9918		9237				
Heterogeneity: Tau^2 = 0.00; Chi^2 = 98.93, df = 24 (P < 0.00001); I^2 = 76%							
Test for overall effect: Z = 5.15 (P < 0.00001)							
Test for subgroup differences: Chi^2 = 3.26, df = 2 (P = 0.20), I^2 = 38.6%							

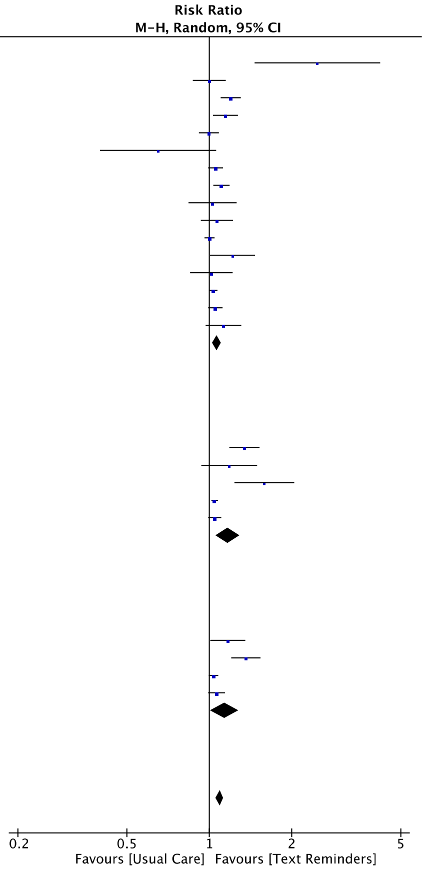

Figure S7: Subgroup analysis based on vaccination type

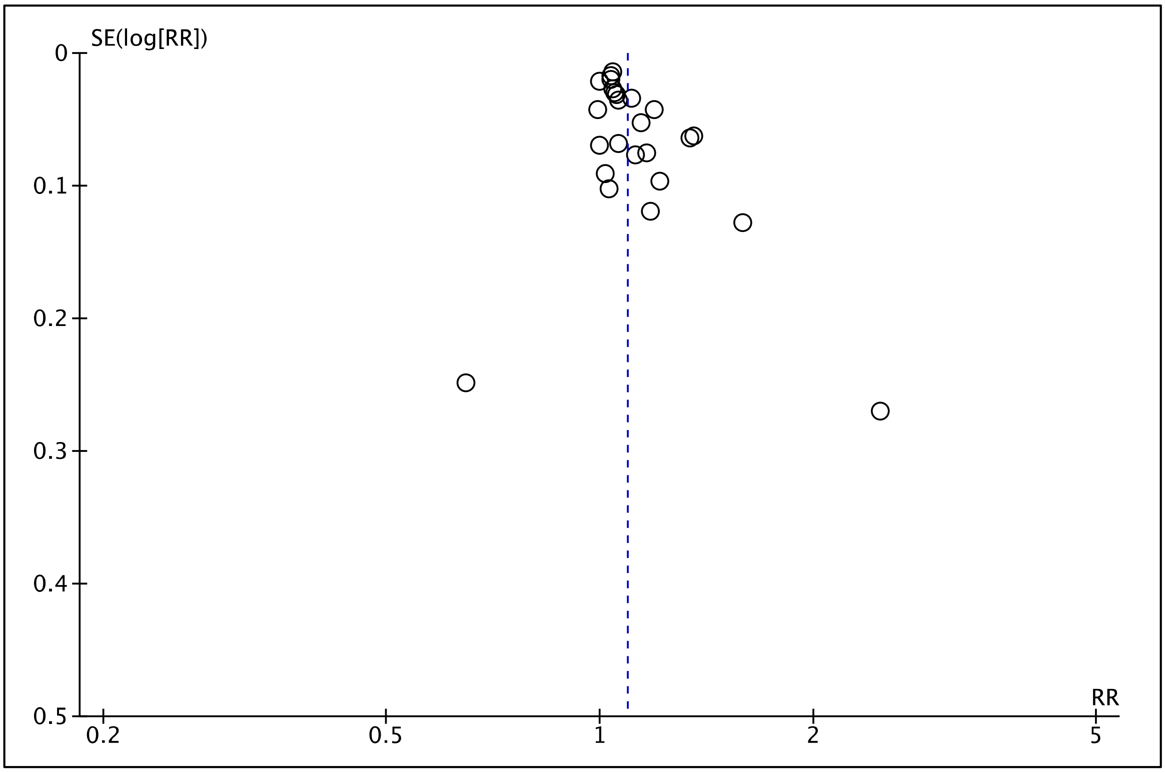

Figure S8: Funnel plot illustrating publication bias.
